# 3-(6-Benz­yloxy-2,2-dimethyl­perhydro­furo[2,3-*d*][1,3]dioxol-5-yl)-5-(4-bromo­phen­yl)-2-phenyl­perhydro­pyrrolo[3,4-*d*]isoxazole-4,6-dione

**DOI:** 10.1107/S1600536809015104

**Published:** 2009-04-30

**Authors:** M. NizamMohideen, M. Damodiran, A. SubbiahPandi, P. T. Perumal

**Affiliations:** a Department of Physics, The New College (Autonomous), Chennai 600 014, India; b Organic Chemistry Division, Central Leather Research Institute, Chennai 600 020, India; c Department of Physics, Presidency College (Autonomous), Chennai 600 005, India

## Abstract

In the title compound, C_31_H_29_BrN_2_O_7_, the isoxazolidine ring adopts a twist conformation, while the tetrahydrofuran, dioxolone and pyrrole rings adopt envelope conformations. The structure is stabilized by inter­molecular C—H⋯O hydrogen bonds and C—H⋯π inter­actions.

## Related literature

For general background to isoxazolidines, see: Ali *et al.* (1988 ▶); Goti *et al.* (1997 ▶); Kumar *et al.* (2003 ▶); Huisgen (1984 ▶). For ring puckering parameters see: Cremer & Pople (1975 ▶); Nardelli (1983 ▶).
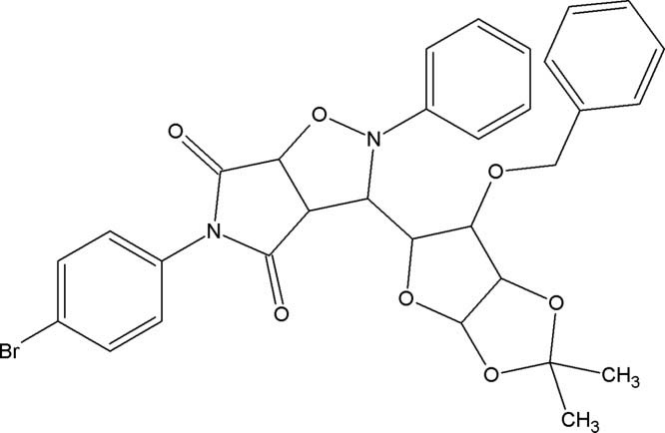


## Experimental

### 

#### Crystal data

C_31_H_29_BrN_2_O_7_
*M* *_r_* = 621.47
Monoclinic, 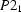

*a* = 15.0680 (12) Å
*b* = 6.6801 (5) Å
*c* = 15.8550 (12) Å
β = 117.578 (2)°
*V* = 1414.57 (19) Å^3^
*Z* = 2
Mo *K*α radiation
μ = 1.51 mm^−1^
*T* = 293 K
0.3 × 0.2 × 0.2 mm

#### Data collection

Bruker Kappa APEXII CCD diffractometer
Absorption correction: multi-scan (*SADABS*; Bruker 2004 ▶) *T*_min_ = 0.734, *T*_max_ = 0.740
20450 measured reflections
9003 independent reflections
5502 reflections with *I* > 2σ(*I*)
*R*_int_ = 0.027

#### Refinement

*R*[*F* ^2^ > 2σ(*F* ^2^)] = 0.042
*wR*(*F* ^2^) = 0.138
*S* = 1.00
9003 reflections
372 parameters
1 restraint
H-atom parameters constrained
Δρ_max_ = 0.66 e Å^−3^
Δρ_min_ = −0.38 e Å^−3^
Absolute structure: Flack (1983 ▶), 3886 Friedel pairs
Flack parameter: −0.001 (8)

### 

Data collection: *APEX2* (Bruker, 2004 ▶); cell refinement: *APEX2* and *SAINT* (Bruker, 2004 ▶); data reduction: *SAINT* and *XPREP* (Bruker, 2004 ▶); program(s) used to solve structure: *SHELXS97* (Sheldrick, 2008 ▶); program(s) used to refine structure: *SHELXL97* (Sheldrick, 2008 ▶); molecular graphics: *ORTEP-3* (Farrugia, 1997 ▶); software used to prepare material for publication: *SHELXL97* and *PLATON* (Spek, 2009 ▶).

## Supplementary Material

Crystal structure: contains datablocks global, I. DOI: 10.1107/S1600536809015104/bt2932sup1.cif

Structure factors: contains datablocks I. DOI: 10.1107/S1600536809015104/bt2932Isup2.hkl

Additional supplementary materials:  crystallographic information; 3D view; checkCIF report

## Figures and Tables

**Table 1:** Hydrogen-bond geometry (Å, °)

*D*—H⋯*A*	*D*—H	H⋯*A*	*D*⋯*A*	*D*—H⋯*A*
C1—H1⋯O2^i^	0.93	2.46	3.273 (3)	145
C5—H5⋯O5^ii^	0.93	2.52	3.198 (3)	130
C9—H9⋯O1^ii^	0.98	2.58	3.418 (3)	144
C19—H19*B*⋯O3^i^	0.97	2.58	3.360 (3)	137
C17—H17b⋯*Cg*1^iii^	0.96	2.86	3.720 (4)	150
C21—H21⋯*Cg*2^iv^	0.93	2.67	3.559 (7)	160

Symmetry codes: (i) 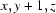; (ii) 
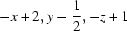
; (iii) 
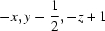
; (iv) 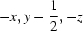. *Cg*1 and *Cg*2 are the centroids of the C1–C6 and C20–C25 rings, respectively.

**Fig. 1.**
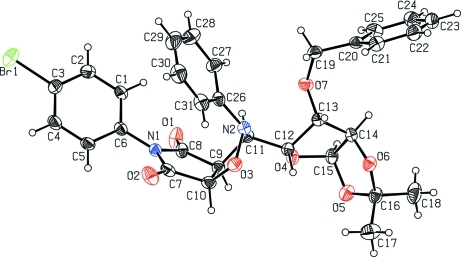
A perspective view of the title compound with the atom-numbering scheme. Displacement ellipsoids are drawn at the 30% probability level.

**Fig. 2.**
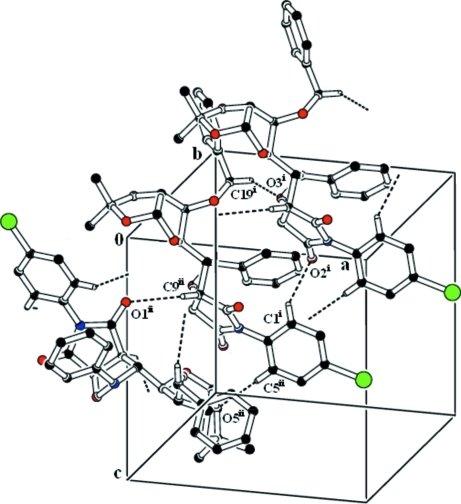
Partial packing view, showing the C—H···O hydrogen-bonding interactions (dashed lines), resulting in the formation of an infinite chain. H atoms not involved in the hydrogen bonding have been omitted for clarity. [Symmetry codes: (i) x, y + 1, z; (ii) -x + 2, y - 1/2, -z + 1.]

## supplementary crystallographic information

### Comment

Isoxazolidines are potential precursors for biologically important compounds such as amino sugars (Ali *et al.*, 1988), alkaloids (Goti *et al.*, 1997), and exhibit antibacterial and antifungal activities (Kumar *et al.*, 2003). The stereochemistry, such as regioselectivity and enantioselectivity, of heterocyclic isoxazole compounds (Huisgen, 1984) can be studied by 1,3-dipolar cycloaddition reactions. In view of these important properties, the crystal structure of the title compound, (I), has been determined.

A perspective view of compound (I) with the atom-numbering scheme is shown in Fig. 1.   The dihedral angle between the phenyl rings C1—C6 and C20—C25, and, C20—C25 and C26—C31, C1—C6 and C26—C31 are 62.4 (1), 75.9 (1) and 42.1 (1)°, respectively.

The five membered isoxazolidine ring (C9-C1,O3,N2) adopts a twisted conformation On O3 and N2 with a pseudo-twofold axis passing through C11-C9 bond. The other five membered tetrahydrofuran, dioxolone and pyrrole rings adopt envelope conformation on C12, O6 and C8 respectively. The puckering parameters (Cremer & Pople, 1975) and the lowest displacement asymmetry parameters (Nardelli, 1983) as follows: for the isoxazolidine ring q_2_ = 0.353 (1) Å, φ = 26.0 (1)°, Δ_S_(N2) is 9.6 (1)° and Δ_2_(C9) is 7.4 (1)°, for the tetrahydrofuran ring q_2_ = 0.395 (1) Å, φ = 313.0 (1)°, Δ_S_(C12) is 6.6 (1)° and Δ_2_(C15) is 11.2 (1)°, for the dioxolone ring q_2_ = 0.232 (1) Å, φ = 295.8 (1)°, Δ_S_(O6) is 5.3 (1)° and Δ_2_(C15) is 6.2 (1)° and for the pyrrole ring q_2_ = 0.085 (1) Å, φ = 140.0 (1)°, Δ_S_(C8) is 0.2 (1)° and Δ_2_(C7) is 4.5 (1)°.

The crystal structure is stabilized by intermolecular C—H···O hydrogen bonds (Table 1; Fig. 2). The crystal structure is further stabilized by C—H···π interactions involing rings C17—H17B···*Cg*1 and C21—H21···*Cg*2 (*Cg*1 and *Cg*2 denote the centroid of the C1—C6 and C20—C25 phenyl rings).

### Experimental

A mixture of D-glucose derived nitrone (0.5 mmol) and maleimide (0.5 mmol) was refluxed in dry toluene (10 ml) until completion of the reaction as evidenced by TLC analysis. The solvent was evaporated under reduced pressure. The crude product was purified by column chromatography on silica gel (Merck, 100–200 mesh, ethylacetate-petroleum ether (1:9). Single crystals of the title compound suitable for X-ray diffraction were obtained by recrystallization from ethanol.

### Refinement

All H atoms were positioned geometrically, with C—H = 0.93–0.98 Å and constrained to ride on their parent atoms, with *U*_iso_(H) = *xU*_eq_(C), where *x* = 1.5 for methyl H and *x* = 1.2 for all other H atoms.

### Crystal data

**Table:**

C_31_H_29_BrN_2_O_7_	*F*(000) = 640
*M**_r_* = 621.47	*D*_x_ = 1.459 Mg m^−^^3^
Monoclinic, *P*2_1_	Mo *K*α radiation, λ = 0.71073 Å
Hall symbol: P 2yb	Cell parameters from 5502 reflections
*a* = 15.0680 (12) Å	θ = 2.5–25°
*b* = 6.6801 (5) Å	µ = 1.51 mm^−^^1^
*c* = 15.8550 (12) Å	*T* = 293 K
β = 117.578 (2)°	Needle, colourless
*V* = 1414.57 (19) Å^3^	0.3 × 0.2 × 0.2 mm
*Z* = 2	

### Data collection

**Table:**

Bruker Kappa APEXII CCD diffractometer	9003 independent reflections
Radiation source: fine-focus sealed tube	5502 reflections with *I* > 2σ(*I*)
graphite	*R*_int_ = 0.027
ω and φ scans	θ_max_ = 31.7°, θ_min_ = 1.5°
Absorption correction: multi-scan (*SADABS*; Bruker 2004)	*h* = −22→22
*T*_min_ = 0.734, *T*_max_ = 0.740	*k* = −9→8
20450 measured reflections	*l* = −23→23

### Refinement

**Table:**

Refinement on *F*^2^	Secondary atom site location: difference Fourier map
Least-squares matrix: full	Hydrogen site location: inferred from neighbouring sites
*R*[*F*^2^ > 2σ(*F*^2^)] = 0.042	H-atom parameters constrained
*wR*(*F*^2^) = 0.138	*w* = 1/[σ^2^(*F*_o_^2^) + (0.066*P*)^2^] where *P* = (*F*_o_^2^ + 2*F*_c_^2^)/3
*S* = 1.00	(Δ/σ)_max_ = 0.001
9003 reflections	Δρ_max_ = 0.66 e Å^−^^3^
372 parameters	Δρ_min_ = −0.38 e Å^−^^3^
1 restraint	Absolute structure: Flack (1983), 3886 Friedel pairs
Primary atom site location: structure-invariant direct methods	Flack parameter: −0.001 (8)

### Special details

**Table:**

Geometry. All e.s.d.'s (except the e.s.d. in the dihedral angle between two l.s. planes) are estimated using the full covariance matrix. The cell e.s.d.'s are taken into account individually in the estimation of e.s.d.'s in distances, angles and torsion angles; correlations between e.s.d.'s in cell parameters are only used when they are defined by crystal symmetry. An approximate (isotropic) treatment of cell e.s.d.'s is used for estimating e.s.d.'s involving l.s. planes.
Refinement. Refinement of *F*^2^ against ALL reflections. The weighted *R*-factor *wR* and goodness of fit *S* are based on *F*^2^, conventional *R*-factors *R* are based on *F*, with *F* set to zero for negative *F*^2^. The threshold expression of *F*^2^ > σ(*F*^2^) is used only for calculating *R*-factors(gt) *etc*. and is not relevant to the choice of reflections for refinement. *R*-factors based on *F*^2^ are statistically about twice as large as those based on *F*, and *R*- factors based on ALL data will be even larger.

### Fractional atomic coordinates and isotropic or equivalent isotropic displacement parameters (Å^2^)

**Table:**

	*x*	*y*	*z*	*U*_iso_*/*U*_eq_	
C1	0.64016 (18)	0.3845 (4)	0.35916 (18)	0.0430 (6)	
H1	0.6865	0.4725	0.4028	0.052*	
C2	0.5464 (2)	0.4517 (5)	0.2949 (2)	0.0524 (7)	
H2	0.5293	0.5853	0.2952	0.063*	
C3	0.4785 (2)	0.3213 (5)	0.2304 (2)	0.0557 (7)	
C4	0.5020 (2)	0.1225 (5)	0.2294 (2)	0.0576 (8)	
H4	0.4553	0.0349	0.1858	0.069*	
C5	0.5958 (2)	0.0545 (4)	0.2940 (2)	0.0462 (6)	
H5	0.6125	−0.0796	0.2942	0.055*	
C6	0.66469 (18)	0.1858 (4)	0.35820 (16)	0.0359 (5)	
C7	0.7771 (2)	−0.0588 (4)	0.47898 (17)	0.0415 (5)	
C8	0.85005 (18)	0.2249 (4)	0.45433 (17)	0.0381 (5)	
C9	0.93114 (18)	0.1327 (4)	0.54302 (17)	0.0370 (5)	
H9	0.9931	0.1105	0.5385	0.044*	
C10	0.88614 (19)	−0.0602 (4)	0.55592 (17)	0.0427 (5)	
H10	0.9220	−0.1772	0.5498	0.051*	
C11	0.94837 (17)	0.2561 (3)	0.63122 (16)	0.0340 (5)	
H11	0.9266	0.3948	0.6132	0.041*	
C12	1.05597 (18)	0.2501 (4)	0.70747 (16)	0.0352 (5)	
H12	1.0775	0.1109	0.7240	0.042*	
C13	1.07749 (18)	0.3678 (3)	0.79726 (16)	0.0347 (5)	
H13	1.0594	0.2926	0.8402	0.042*	
C14	1.18993 (18)	0.3982 (4)	0.83916 (16)	0.0379 (5)	
H14	1.2135	0.5202	0.8776	0.045*	
C15	1.20853 (17)	0.3989 (4)	0.75221 (16)	0.0402 (5)	
H15	1.2319	0.5306	0.7437	0.048*	
C16	1.3149 (2)	0.1722 (4)	0.86285 (19)	0.0447 (6)	
C17	1.3205 (3)	−0.0535 (6)	0.8586 (3)	0.0690 (9)	
H17A	1.3361	−0.1092	0.9197	0.104*	
H17B	1.3718	−0.0898	0.8415	0.104*	
H17C	1.2572	−0.1047	0.8118	0.104*	
C18	1.4135 (2)	0.2643 (7)	0.9303 (3)	0.0759 (11)	
H18A	1.4063	0.4071	0.9304	0.114*	
H18B	1.4629	0.2314	0.9104	0.114*	
H18C	1.4342	0.2134	0.9933	0.114*	
C19	0.9839 (2)	0.6190 (4)	0.82909 (19)	0.0432 (6)	
H19A	0.9439	0.5111	0.8349	0.052*	
H19B	0.9392	0.7302	0.7980	0.052*	
C20	1.05951 (18)	0.6836 (3)	0.92725 (17)	0.0359 (5)	
C21	1.0676 (2)	0.5854 (4)	1.00695 (19)	0.0453 (6)	
H21	1.0286	0.4729	1.0005	0.054*	
C22	1.1336 (2)	0.6541 (5)	1.0960 (2)	0.0557 (7)	
H22	1.1379	0.5884	1.1495	0.067*	
C23	1.1925 (2)	0.8162 (5)	1.1073 (2)	0.0600 (8)	
H23	1.2366	0.8609	1.1680	0.072*	
C24	1.1866 (2)	0.9139 (5)	1.0285 (2)	0.0587 (7)	
H24	1.2271	1.0242	1.0356	0.070*	
C25	1.1202 (2)	0.8473 (4)	0.9390 (2)	0.0471 (6)	
H25	1.1162	0.9134	0.8857	0.057*	
C26	0.78715 (19)	0.2237 (4)	0.63967 (16)	0.0380 (5)	
C27	0.7611 (2)	0.4255 (5)	0.6230 (2)	0.0486 (6)	
H27	0.8081	0.5200	0.6270	0.058*	
C28	0.6640 (3)	0.4843 (6)	0.6006 (3)	0.0656 (9)	
H28	0.6465	0.6187	0.5886	0.079*	
C29	0.5940 (2)	0.3490 (6)	0.5957 (3)	0.0699 (10)	
H29	0.5294	0.3901	0.5807	0.084*	
C30	0.6209 (2)	0.1490 (6)	0.6135 (2)	0.0645 (9)	
H30	0.5737	0.0551	0.6100	0.077*	
C31	0.7164 (2)	0.0883 (5)	0.6361 (2)	0.0511 (7)	
H31	0.7336	−0.0460	0.6492	0.061*	
N1	0.76087 (15)	0.1169 (3)	0.42650 (14)	0.0342 (4)	
N2	0.88766 (16)	0.1595 (3)	0.66968 (14)	0.0367 (4)	
O1	0.85721 (15)	0.3700 (3)	0.41335 (14)	0.0592 (6)	
O2	0.71522 (18)	−0.1839 (3)	0.46666 (16)	0.0608 (6)	
O3	0.89191 (14)	−0.0499 (3)	0.64921 (12)	0.0442 (4)	
O4	1.11759 (13)	0.3476 (3)	0.67218 (12)	0.0419 (4)	
O5	1.28173 (14)	0.2528 (3)	0.77001 (14)	0.0501 (5)	
O6	1.23964 (13)	0.2227 (3)	0.88938 (12)	0.0419 (4)	
O7	1.02692 (15)	0.5529 (3)	0.77010 (13)	0.0429 (4)	
Br1	0.35138 (3)	0.41736 (8)	0.14395 (3)	0.1005 (2)	

### Atomic displacement parameters (Å^2^)

**Table:**

	*U*^11^	*U*^22^	*U*^33^	*U*^12^	*U*^13^	*U*^23^
C1	0.0417 (13)	0.0400 (13)	0.0428 (13)	−0.0072 (11)	0.0157 (11)	−0.0124 (11)
C2	0.0489 (15)	0.0478 (14)	0.0581 (17)	0.0043 (12)	0.0228 (13)	−0.0084 (13)
C3	0.0343 (14)	0.0731 (19)	0.0541 (18)	0.0086 (13)	0.0158 (13)	−0.0138 (14)
C4	0.0374 (14)	0.0683 (19)	0.0611 (18)	−0.0091 (13)	0.0177 (13)	−0.0319 (15)
C5	0.0384 (13)	0.0423 (12)	0.0578 (17)	−0.0065 (11)	0.0222 (12)	−0.0192 (12)
C6	0.0352 (12)	0.0398 (12)	0.0353 (12)	−0.0065 (9)	0.0185 (10)	−0.0070 (9)
C7	0.0558 (15)	0.0311 (11)	0.0400 (13)	−0.0054 (11)	0.0244 (11)	−0.0081 (10)
C8	0.0340 (12)	0.0461 (13)	0.0338 (12)	−0.0050 (10)	0.0153 (10)	−0.0001 (10)
C9	0.0329 (12)	0.0425 (12)	0.0357 (12)	0.0009 (9)	0.0161 (10)	0.0006 (10)
C10	0.0530 (14)	0.0315 (11)	0.0431 (14)	0.0070 (11)	0.0218 (11)	0.0019 (10)
C11	0.0334 (11)	0.0352 (11)	0.0328 (11)	0.0030 (9)	0.0149 (9)	0.0044 (9)
C12	0.0341 (12)	0.0363 (11)	0.0350 (12)	−0.0004 (9)	0.0158 (10)	0.0014 (9)
C13	0.0381 (12)	0.0349 (11)	0.0329 (11)	0.0004 (9)	0.0181 (10)	0.0045 (9)
C14	0.0393 (11)	0.0383 (11)	0.0316 (11)	−0.0032 (10)	0.0127 (9)	−0.0022 (10)
C15	0.0358 (11)	0.0427 (12)	0.0385 (12)	−0.0072 (10)	0.0142 (10)	0.0027 (11)
C16	0.0345 (13)	0.0587 (15)	0.0402 (13)	0.0000 (11)	0.0166 (11)	0.0047 (12)
C17	0.076 (2)	0.0624 (19)	0.080 (2)	0.0156 (18)	0.0458 (19)	0.0102 (17)
C18	0.0406 (18)	0.111 (3)	0.064 (2)	−0.0090 (18)	0.0140 (16)	−0.008 (2)
C19	0.0424 (14)	0.0470 (14)	0.0435 (14)	0.0091 (11)	0.0227 (12)	0.0013 (11)
C20	0.0380 (12)	0.0380 (12)	0.0371 (12)	0.0077 (10)	0.0219 (10)	0.0018 (10)
C21	0.0496 (15)	0.0464 (14)	0.0456 (15)	0.0005 (12)	0.0268 (13)	0.0069 (11)
C22	0.0602 (18)	0.0670 (18)	0.0401 (15)	0.0136 (15)	0.0234 (14)	0.0121 (13)
C23	0.0455 (16)	0.077 (2)	0.0481 (17)	0.0061 (15)	0.0141 (13)	−0.0170 (15)
C24	0.0553 (16)	0.0568 (16)	0.0696 (19)	−0.0134 (15)	0.0337 (14)	−0.0139 (16)
C25	0.0585 (17)	0.0401 (13)	0.0524 (16)	−0.0017 (11)	0.0338 (14)	0.0016 (11)
C26	0.0387 (13)	0.0487 (13)	0.0282 (11)	−0.0048 (10)	0.0167 (10)	−0.0032 (10)
C27	0.0428 (13)	0.0461 (14)	0.0596 (16)	0.0018 (12)	0.0259 (12)	0.0012 (13)
C28	0.0530 (19)	0.071 (2)	0.078 (2)	0.0156 (15)	0.0347 (17)	0.0065 (17)
C29	0.0366 (15)	0.108 (3)	0.067 (2)	0.0044 (16)	0.0253 (15)	−0.008 (2)
C30	0.0486 (18)	0.088 (3)	0.065 (2)	−0.0179 (17)	0.0339 (16)	−0.0119 (18)
C31	0.0508 (16)	0.0582 (16)	0.0496 (16)	−0.0155 (13)	0.0278 (13)	−0.0062 (13)
N1	0.0341 (10)	0.0343 (9)	0.0339 (10)	−0.0038 (7)	0.0155 (8)	−0.0032 (8)
N2	0.0378 (10)	0.0365 (10)	0.0355 (10)	−0.0027 (8)	0.0166 (8)	−0.0001 (8)
O1	0.0442 (10)	0.0710 (13)	0.0481 (11)	−0.0187 (9)	0.0092 (8)	0.0213 (10)
O2	0.0768 (15)	0.0417 (10)	0.0591 (13)	−0.0238 (10)	0.0274 (12)	−0.0086 (9)
O3	0.0540 (10)	0.0345 (8)	0.0416 (9)	0.0038 (8)	0.0198 (8)	0.0092 (7)
O4	0.0365 (9)	0.0575 (11)	0.0325 (8)	−0.0067 (8)	0.0167 (7)	−0.0015 (7)
O5	0.0431 (11)	0.0668 (12)	0.0457 (10)	0.0046 (9)	0.0250 (9)	0.0085 (9)
O6	0.0403 (10)	0.0494 (10)	0.0364 (9)	0.0036 (8)	0.0180 (8)	0.0072 (7)
O7	0.0575 (11)	0.0388 (9)	0.0350 (9)	0.0079 (8)	0.0235 (8)	0.0037 (7)
Br1	0.0491 (2)	0.1229 (4)	0.0909 (3)	0.0303 (2)	−0.00047 (17)	−0.0247 (3)

### Geometric parameters (Å, °)

**Table:**

C1—C2	1.379 (4)	C16—O6	1.421 (3)
C1—C6	1.380 (4)	C16—O5	1.423 (3)
C1—H1	0.9300	C16—C18	1.502 (4)
C2—C3	1.372 (4)	C16—C17	1.513 (5)
C2—H2	0.9300	C17—H17A	0.9600
C3—C4	1.377 (5)	C17—H17B	0.9600
C3—Br1	1.878 (3)	C17—H17C	0.9600
C4—C5	1.384 (4)	C18—H18A	0.9600
C4—H4	0.9300	C18—H18B	0.9600
C5—C6	1.380 (3)	C18—H18C	0.9600
C5—H5	0.9300	C19—O7	1.433 (3)
C6—N1	1.425 (3)	C19—C20	1.505 (4)
C7—O2	1.198 (3)	C19—H19A	0.9700
C7—N1	1.393 (3)	C19—H19B	0.9700
C7—C10	1.527 (4)	C20—C21	1.378 (3)
C8—O1	1.199 (3)	C20—C25	1.382 (4)
C8—N1	1.405 (3)	C21—C22	1.376 (4)
C8—C9	1.503 (3)	C21—H21	0.9300
C9—C10	1.513 (4)	C22—C23	1.359 (5)
C9—C11	1.538 (3)	C22—H22	0.9300
C9—H9	0.9800	C23—C24	1.375 (5)
C10—O3	1.443 (3)	C23—H23	0.9300
C10—H10	0.9800	C24—C25	1.377 (4)
C11—N2	1.464 (3)	C24—H24	0.9300
C11—C12	1.509 (3)	C25—H25	0.9300
C11—H11	0.9800	C26—C31	1.379 (4)
C12—O4	1.442 (3)	C26—C27	1.395 (4)
C12—C13	1.524 (3)	C26—N2	1.427 (3)
C12—H12	0.9800	C27—C28	1.392 (4)
C13—O7	1.411 (3)	C27—H27	0.9300
C13—C14	1.520 (3)	C28—C29	1.364 (5)
C13—H13	0.9800	C28—H28	0.9300
C14—O6	1.420 (3)	C29—C30	1.386 (6)
C14—C15	1.529 (3)	C29—H29	0.9300
C14—H14	0.9800	C30—C31	1.374 (4)
C15—O5	1.400 (3)	C30—H30	0.9300
C15—O4	1.413 (3)	C31—H31	0.9300
C15—H15	0.9800	N2—O3	1.444 (3)
			
C2—C1—C6	119.5 (2)	O6—C16—C17	108.6 (2)
C2—C1—H1	120.2	O5—C16—C17	109.3 (3)
C6—C1—H1	120.2	C18—C16—C17	112.5 (3)
C3—C2—C1	120.0 (3)	C16—C17—H17A	109.5
C3—C2—H2	120.0	C16—C17—H17B	109.5
C1—C2—H2	120.0	H17A—C17—H17B	109.5
C2—C3—C4	120.9 (3)	C16—C17—H17C	109.5
C2—C3—Br1	119.0 (2)	H17A—C17—H17C	109.5
C4—C3—Br1	120.2 (2)	H17B—C17—H17C	109.5
C3—C4—C5	119.3 (3)	C16—C18—H18A	109.5
C3—C4—H4	120.4	C16—C18—H18B	109.5
C5—C4—H4	120.4	H18A—C18—H18B	109.5
C6—C5—C4	119.9 (3)	C16—C18—H18C	109.5
C6—C5—H5	120.1	H18A—C18—H18C	109.5
C4—C5—H5	120.1	H18B—C18—H18C	109.5
C1—C6—C5	120.4 (2)	O7—C19—C20	114.1 (2)
C1—C6—N1	119.1 (2)	O7—C19—H19A	108.7
C5—C6—N1	120.5 (2)	C20—C19—H19A	108.7
O2—C7—N1	125.6 (3)	O7—C19—H19B	108.7
O2—C7—C10	126.5 (2)	C20—C19—H19B	108.7
N1—C7—C10	107.9 (2)	H19A—C19—H19B	107.6
O1—C8—N1	123.9 (2)	C21—C20—C25	118.8 (2)
O1—C8—C9	126.9 (2)	C21—C20—C19	121.0 (2)
N1—C8—C9	109.2 (2)	C25—C20—C19	120.2 (2)
C8—C9—C10	104.74 (19)	C22—C21—C20	119.8 (3)
C8—C9—C11	110.7 (2)	C22—C21—H21	120.1
C10—C9—C11	103.35 (19)	C20—C21—H21	120.1
C8—C9—H9	112.5	C23—C22—C21	121.2 (3)
C10—C9—H9	112.5	C23—C22—H22	119.4
C11—C9—H9	112.5	C21—C22—H22	119.4
O3—C10—C9	106.3 (2)	C22—C23—C24	119.7 (3)
O3—C10—C7	110.5 (2)	C22—C23—H23	120.2
C9—C10—C7	105.72 (19)	C24—C23—H23	120.2
O3—C10—H10	111.4	C25—C24—C23	119.5 (3)
C9—C10—H10	111.4	C25—C24—H24	120.2
C7—C10—H10	111.4	C23—C24—H24	120.2
N2—C11—C12	107.55 (18)	C24—C25—C20	120.9 (3)
N2—C11—C9	105.52 (19)	C24—C25—H25	119.5
C12—C11—C9	112.38 (19)	C20—C25—H25	119.5
N2—C11—H11	110.4	C31—C26—C27	118.8 (3)
C12—C11—H11	110.4	C31—C26—N2	119.8 (2)
C9—C11—H11	110.4	C27—C26—N2	121.1 (2)
O4—C12—C11	108.86 (18)	C28—C27—C26	119.5 (3)
O4—C12—C13	103.47 (18)	C28—C27—H27	120.3
C11—C12—C13	114.83 (19)	C26—C27—H27	120.3
O4—C12—H12	109.8	C29—C28—C27	121.3 (3)
C11—C12—H12	109.8	C29—C28—H28	119.3
C13—C12—H12	109.8	C27—C28—H28	119.3
O7—C13—C14	110.6 (2)	C28—C29—C30	118.8 (3)
O7—C13—C12	108.39 (18)	C28—C29—H29	120.6
C14—C13—C12	100.91 (18)	C30—C29—H29	120.6
O7—C13—H13	112.1	C31—C30—C29	120.6 (3)
C14—C13—H13	112.1	C31—C30—H30	119.7
C12—C13—H13	112.1	C29—C30—H30	119.7
O6—C14—C13	109.06 (19)	C30—C31—C26	120.9 (3)
O6—C14—C15	103.8 (2)	C30—C31—H31	119.5
C13—C14—C15	103.82 (17)	C26—C31—H31	119.5
O6—C14—H14	113.1	C7—N1—C8	111.6 (2)
C13—C14—H14	113.1	C7—N1—C6	124.2 (2)
C15—C14—H14	113.1	C8—N1—C6	124.1 (2)
O5—C15—O4	111.0 (2)	C26—N2—O3	111.51 (19)
O5—C15—C14	105.81 (19)	C26—N2—C11	120.00 (19)
O4—C15—C14	107.62 (18)	O3—N2—C11	103.42 (17)
O5—C15—H15	110.8	N2—O3—C10	106.78 (17)
O4—C15—H15	110.8	C15—O4—C12	106.98 (17)
C14—C15—H15	110.8	C15—O5—C16	109.83 (19)
O6—C16—O5	105.7 (2)	C14—O6—C16	108.42 (18)
O6—C16—C18	110.7 (2)	C13—O7—C19	114.50 (18)
O5—C16—C18	109.9 (3)		
			
C6—C1—C2—C3	−0.2 (4)	C19—C20—C25—C24	177.0 (3)
C1—C2—C3—C4	0.7 (5)	C31—C26—C27—C28	−1.9 (4)
C1—C2—C3—Br1	179.9 (2)	N2—C26—C27—C28	−175.4 (3)
C2—C3—C4—C5	−0.5 (5)	C26—C27—C28—C29	0.9 (5)
Br1—C3—C4—C5	−179.7 (2)	C27—C28—C29—C30	−0.1 (6)
C3—C4—C5—C6	−0.3 (5)	C28—C29—C30—C31	0.3 (6)
C2—C1—C6—C5	−0.5 (4)	C29—C30—C31—C26	−1.4 (5)
C2—C1—C6—N1	−178.4 (2)	C27—C26—C31—C30	2.2 (4)
C4—C5—C6—C1	0.8 (4)	N2—C26—C31—C30	175.7 (3)
C4—C5—C6—N1	178.6 (2)	O2—C7—N1—C8	−175.9 (2)
O1—C8—C9—C10	−172.5 (3)	C10—C7—N1—C8	5.6 (3)
N1—C8—C9—C10	9.1 (3)	O2—C7—N1—C6	8.6 (4)
O1—C8—C9—C11	76.7 (3)	C10—C7—N1—C6	−169.94 (19)
N1—C8—C9—C11	−101.7 (2)	O1—C8—N1—C7	172.1 (2)
C8—C9—C10—O3	−123.0 (2)	C9—C8—N1—C7	−9.4 (3)
C11—C9—C10—O3	−7.1 (2)	O1—C8—N1—C6	−12.3 (4)
C8—C9—C10—C7	−5.5 (2)	C9—C8—N1—C6	166.1 (2)
C11—C9—C10—C7	110.4 (2)	C1—C6—N1—C7	134.2 (2)
O2—C7—C10—O3	−63.6 (3)	C5—C6—N1—C7	−43.7 (3)
N1—C7—C10—O3	114.9 (2)	C1—C6—N1—C8	−40.8 (3)
O2—C7—C10—C9	−178.2 (3)	C5—C6—N1—C8	141.3 (2)
N1—C7—C10—C9	0.3 (2)	C31—C26—N2—O3	27.9 (3)
C8—C9—C11—N2	95.2 (2)	C27—C26—N2—O3	−158.7 (2)
C10—C9—C11—N2	−16.4 (2)	C31—C26—N2—C11	149.0 (2)
C8—C9—C11—C12	−147.9 (2)	C27—C26—N2—C11	−37.7 (3)
C10—C9—C11—C12	100.5 (2)	C12—C11—N2—C26	148.6 (2)
N2—C11—C12—O4	−179.02 (17)	C9—C11—N2—C26	−91.2 (2)
C9—C11—C12—O4	65.3 (2)	C12—C11—N2—O3	−86.4 (2)
N2—C11—C12—C13	−63.6 (2)	C9—C11—N2—O3	33.8 (2)
C9—C11—C12—C13	−179.3 (2)	C26—N2—O3—C10	91.0 (2)
O4—C12—C13—O7	75.3 (2)	C11—N2—O3—C10	−39.3 (2)
C11—C12—C13—O7	−43.2 (3)	C9—C10—O3—N2	28.7 (2)
O4—C12—C13—C14	−40.9 (2)	C7—C10—O3—N2	−85.5 (2)
C11—C12—C13—C14	−159.34 (19)	O5—C15—O4—C12	95.9 (2)
O7—C13—C14—O6	163.81 (18)	C14—C15—O4—C12	−19.5 (3)
C12—C13—C14—O6	−81.6 (2)	C11—C12—O4—C15	160.8 (2)
O7—C13—C14—C15	−86.0 (2)	C13—C12—O4—C15	38.2 (2)
C12—C13—C14—C15	28.5 (2)	O4—C15—O5—C16	−120.5 (2)
O6—C14—C15—O5	−11.6 (2)	C14—C15—O5—C16	−4.1 (3)
C13—C14—C15—O5	−125.6 (2)	O6—C16—O5—C15	18.4 (3)
O6—C14—C15—O4	107.1 (2)	C18—C16—O5—C15	−101.0 (3)
C13—C14—C15—O4	−6.9 (3)	C17—C16—O5—C15	135.0 (3)
O7—C19—C20—C21	−118.0 (3)	C13—C14—O6—C16	133.4 (2)
O7—C19—C20—C25	64.0 (3)	C15—C14—O6—C16	23.3 (2)
C25—C20—C21—C22	1.6 (4)	O5—C16—O6—C14	−26.3 (3)
C19—C20—C21—C22	−176.4 (2)	C18—C16—O6—C14	92.6 (3)
C20—C21—C22—C23	−1.0 (4)	C17—C16—O6—C14	−143.4 (2)
C21—C22—C23—C24	−0.1 (5)	C14—C13—O7—C19	−109.0 (2)
C22—C23—C24—C25	0.6 (5)	C12—C13—O7—C19	141.2 (2)
C23—C24—C25—C20	0.0 (4)	C20—C19—O7—C13	70.5 (3)
C21—C20—C25—C24	−1.1 (4)		

### Hydrogen-bond geometry (Å, °)

**Table:**

*D*—H···*A*	*D*—H	H···*A*	*D*···*A*	*D*—H···*A*
C1—H1···O2^i^	0.93	2.46	3.273 (3)	145
C5—H5···O5^ii^	0.93	2.52	3.198 (3)	130
C9—H9···O1^ii^	0.98	2.58	3.418 (3)	144
C19—H19B···O3^i^	0.97	2.58	3.360 (3)	137
C17—H17b···Cg1^iii^	0.96	2.86	3.720 (4)	150
C21—H21···Cg2^iv^	0.93	2.67	3.559 (7)	160

Symmetry codes: (i) *x*, *y*+1, *z*; (ii) −*x*+2, *y*−1/2, −*z*+1; (iii) −*x*, *y*−1/2, −*z*+1; (iv) −*x*, *y*−1/2, −*z*.
